# Routine health check-ups for adolescents in Mwanza City, Tanzania: stakeholders’ recommendations on its content, venue, and mode of delivery

**DOI:** 10.1186/s12889-023-15956-6

**Published:** 2023-05-30

**Authors:** Yovitha Sedekia, Gerry Mshana, Mussa K. Nsanya, Kid Kohl, Mwita Wambura, Heiner Grosskurth, David A. Ross, Saidi Kapiga

**Affiliations:** 1 Mwanza Intervention Trials Unit, National Institute for Medical Research, Mwanza, Tanzania; 2 The Global Fund to Fight AIDS, TB and Malaria, Geneva, Switzerland; 3 Department of Infectious Disease Epidemiology, London School of Hygiene and Tropical Medicine, London, UK; 4 Institute for Life Course Health Research, Stellenbosch University, Stellenbosch, South Africa

**Keywords:** Adolescents, Health check-up, Routine check-up, Health screening, Stakeholders, Health interventions, Tanzania

## Abstract

**Background:**

Routine adolescent health screening aiming at the detection of unnoticed medical problems may increase awareness among policy makers and contribute to improved health in this population. Research is needed to inform the World Health Organization (WHO) and national health programs to provide evidence-based guidance on whether public health systems should offer comprehensive adolescent health screening, what should be included in different contexts, and how it should be delivered. We conducted formative research to define the content and delivery strategies for health check-ups to be performed in young (10–14 years) and older (15–19 years) adolescents, and to assess whether such services are likely to be acceptable and feasible in Tanzania.

**Methods:**

As part of a collaborative research program coordinated by WHO in Chitungwiza, Zimbabwe; Mwanza City, Tanzania; and Cape Coast, Ghana a series of key informant interviews were conducted from April to July 2020, using a semi-structured guide with purposively selected stakeholders from government departments, non-governmental and community-based organisations, schools and health facilities. Data transcripts were coded using NVivo 12 software and thematic analysis was performed.

**Results:**

We report results from 31 key informant interviews to address four main domains: proposed health conditions for routine health check-ups, health interventions to be combined with such check-ups, preferable venues, and the mode for delivering such screening activities. Stakeholders were supportive of introducing routine health check-ups among adolescents. They recommended focusing on non-communicable diseases, physical disabilities, common mental health problems, reproductive health problems, specific communicable diseases, and hygiene-related problems. They also recommended combining counselling and family planning information with these check-ups. Three venues were proposed: schools, community settings (to reach out-of-school adolescents), and youth-friendly health facilities (for conditions requiring a high level of confidentiality).

**Conclusions:**

Stakeholders were supportive of the proposed routine health check-ups for adolescents, recommending specific health conditions to be screened for in both community and school settings. Based on the above, we plan to conduct implementation research to determine the number of new treatable conditions detected, and the costs of offering such services. In the longer term, evaluation of their health impact and cost-effectiveness will be required to guide policy.

**Supplementary Information:**

The online version contains supplementary material available at 10.1186/s12889-023-15956-6.

## Introduction

The health of adolescents and young adults is an emerging global priority, especially in low- and middle-income countries (LMICs) where 90% of the global population of this age group lives [1]. This high proportion of adolescents has the potential for the ‘demographic dividend’ from accelerated economic growth resulting from the higher workforce. However, adolescents must remain healthy, educated, skilled, and stay or work for their countries for the dividend to be realised. Adolescence is a crucial time for prevention, as exposures to risk factors for communicable and non-communicable conditions often commence during this period, and as behavioural patterns are established that may lead to diseases later in life [2]. The substantial burden of disease among adolescents and consequentially among young adults undermines socio-economic development, particularly in LMICs where accessible and affordable health services for this population are few and often not easy to reach [3]. In the few health systems where such services are available, they are heavily focused on curative services and are generally not well-equipped to detect and provide early treatment and preventive or promotive services for this population [4]. As a result, most adolescents do not seek health care services [5].

Tanzania is home to about 10 million adolescents, accounting for 23% of the total population [6]. Major health problems affecting adolescents in Tanzania include poor sexual and reproductive health including sexually transmitted infections and unintended pregnancies, malnutrition and anaemia, substance abuse, mental health problems, unintentional injuries, and violence including gender-based violence [7–11]. Health services for these major health problems are available in Mwanza city, including primary health facilities, district hospitals, a referral regional hospital and a tertiary hospital for specialised services. To address the health problems of adolescents, the Tanzanian government implemented a five-year National Adolescent Health and Development Strategy (2018–2022) which aimed to strengthen both demand and supply and create an enabling policy environment [7]. It is recognised that limited service delivery points, an inadequate number of suitably trained human resources, and weak supply chains are major barriers to the provision of adolescent-friendly health services which are a critical component of the strategy [7]. In addition, whilst schools can be an optimal venue for vital health information and services to the 98% and 32% of adolescents who are enrolled in primary and secondary schools, respectively, such strategy excludes older adolescents or those who are out of the school system [12, 13].

The effectiveness of routine health check-ups for children and older adults is well established [14, 15] and the World Health Organization (WHO) has issued clear guidelines for national public health systems to provide health check-ups in these populations [16]. Comprehensive adolescent health screening may increase attention to adolescent health issues in settings where dedicated facility-based services are lacking, and could contribute to improved health in this population [17]. However, there has been little research on the potential value of regular check-ups among adolescents and their effectiveness remains unknown [18]. Further research would be needed to assist World Health Organization (WHO) and national health programs in providing evidence-based guidance on whether national public health systems should offer comprehensive adolescent health check-ups, what it should include in different contexts, and how it could be delivered.

We conducted formative research to contribute the knowledge required for the design of adolescent health check-ups in Tanzania. This study is part of a collaborative research program (Y-Check) coordinated by the WHO and involving three African cities: Chitungwiza, Zimbabwe; Mwanza city, Tanzania; and Cape Coast, Ghana [5]. The program comprises formative research and implementation research phases. During the formative research phase which is reported here, we aimed to define the content and delivery strategies for health check-ups among young (10–14 years of age) and older (15–19 years of age) adolescents, and to assess whether such services are likely to be acceptable in Tanzania. Our ultimate aim is to provide evidence on whether adolescent health check-ups should be part of routine health services in the study settings and to evaluate their effectiveness, costs, and cost-effectiveness.

## Methods

### Study setting

We conducted this study between April and July 2020 in Mwanza city on the southern shores of Lake Victoria. Mwanza city is the second largest city in Tanzania and has an estimated population of over 900,000 people [19]. It has 33 public health facilities which include a tertiary/teaching hospital, a regional hospital, 2 district hospitals, 5 health centres, and 24 dispensaries [20, 21]. There are 81 public primary schools and 30 public secondary schools [21]. The primary economic activities in Mwanza city include fishing and industrial fish processing for export markets, agriculture, and large and small-scale mining of gold and diamonds [20, 21].

### Study design and sampling

This was a cross-sectional study using a semi-structured guide. Researchers with training and experience in qualitative methods conducted a total of 31 in-depth interviews (IDIs) with key informants (Table 1). Participants were selected by purposive sampling from the following groups: heads (or representatives) of selected departments/units under the Ministry of Health (n = 13); Ministry of Education staff coordinating the school health program (n = 1); heads (or representatives) of non-governmental organisations (NGOs) and of community-based organisations (CBOs) providing health services and running programs for adolescents within Mwanza city (n = 9); head teachers (or their deputy) and other teachers responsible for health education from public and private primary schools in Mwanza city (n = 4); and senior health care workers from public and private health facilities that serve the catchment area for each of the four selected schools (n = 3). Twenty- five interviews were conducted face-to-face and six through virtual platforms (such as ©Skype or/and ©Zoom) or by mobile phones.

All invited participants were available except two (a stakeholder from the Ministry of Health and a senior health worker from a private health facility). We conducted 10 in-depth interviews (IDIs) from NGOs/CBOs instead of the nine that were originally planned because at one NGO we interviewed two stakeholders separately; one responsible for project activities at the national level and another at the community level. We conducted four IDIs from two public primary schools (two per school) and two paired IDIs from two private schools (one per school) because the head teacher and the teacher responsible for health education at each school were available at the time of the interviews. Of the three sampled health facilities (one private and two public facilities), we conducted two IDIs, both from public health facilities.

**Table 1:** Sampled institutions and number and type of respondents

Stakeholder’s institution	Stakeholder’s role	Number of Interviews	Remarks
Government departments in the:			
Ministry of Health	Heads or representatives of selected departments/ units^**¥**^	12	One was not available for the interview
Ministry of Education	Head of school health programme	1	
Non-governmental organisations (NGOs) or community-based organisations (CBOs)^**£**^	Heads or representatives	10	We interviewed two stakeholders in one NGO (one at community and another at national level project activities)
Schools	Head teachers and other teacher responsible for health issues		
Public		4	2 schools but separate interviews for head teachers and other teachers responsible for health education at school
Private		2	Paired interviews per school for head teachers and other teachers responsible for health education at school
Health facilities	Head of health facility		
Public		2	
Private*		0	
**Total interviews**		**31**	

^**¥**^ *Departments/units (National malaria control program; national TB program; regional medical officer-Mwanza; sexual and reproductive health department; Tanzania food and nutrition centre; non-communicable disease prevention and control program; social welfare office; national Human Immunodeficiency Virus/ Acquired Immunodeficiency Syndrome (HIV/AIDs) control program; neglected tropical diseases program; mental health and prevention of substance abuse program; national vaccination program; and public health promotion program*

^***£***^ *NGOs/CBOs (Africa Medical and Research Foundation; Tanzania Youth Alliance; Family Health International Tanzania; United Nations Children’s Fund; Kiota Women Health and Development; Johns Hopkins Program for International Education in Gynaecology and Obstetrics; Population Services International Tanzania; The United Nations Population Fund; and PACT Tanzania*

^*^ *Participant not available for interview*

### Data collection and quality control

The topic guide contained questions on the availability of adolescent health services in Mwanza city, existing routine health check-ups, and stakeholders’ recommendations on the health conditions which could be checked for, venues to be used, and mode of delivering the check-ups. The guide was developed in English and then translated into Swahili (Additional files 1 and 2), the national language of Tanzania, and back-translated for accuracy by an independent translator who was fluent in both languages. The interviews were carried out in person or through virtual platforms. During in-person interviews, we implemented standard prevention measures against Covid-19 transmission, including wearing face masks, hand washing, use of hand sanitisers, and keeping the recommended social distance [22]. All interviews were conducted in an environment that ensured privacy and at a time convenient for the stakeholders. The in-person interviews were conducted in a location selected by the stakeholder, most often at the stakeholder´s office. Stakeholders were free to invite another member of staff whom they thought could provide additional information to join the interview.

Three research assistants (one male and two females) with past training and experience in conducting qualitative interviews received a three-day specific training on how to conduct the interviews, and this was followed by two days of pilot testing including how to use the interview guide, and the virtual platforms and mobile phones. All interviews were recorded using a digital audio or smartphone recorder and lasted between 30 and 80 min.

### Data management and analysis

Interviews and field notes were immediately reviewed by the research team after each interview to explore whether there were any new emerging themes or missing information. After each interview, audio recordings were immediately transferred to a password-protected computer and a server at the office of the Mwanza Intervention Trials Unit (MITU) in Mwanza city. The recordings were transcribed verbatim. The transcripts were coded using NVivo 12 software (QSR - International Pty Ltd, Melbourne, Australia) [23]. Quotes included in this manuscript were translated into English. For data analysis, we applied the thematic analysis method [24]. Where the finding was not reported by all interviewed stakeholders, we used non-specific terms such as ‘a few’, ‘several’, ‘some’, or ‘many’ for the semi-quantification [25].

## Results

The results from the 31 key informant interviews are reported on four main domains: health conditions proposed for routine health check-ups, health interventions to be combined with the check-ups, proposed venue, and mode of delivery.

### Health conditions proposed for routine health check-ups

All stakeholders were generally supportive of routine health check-ups for adolescents and six broad categories of health conditions were proposed as part of the check-ups. This included non-communicable diseases (NCDs), physical disabilities, common mental health problems, reproductive health problems, other communicable diseases (CDs) and hygiene related problems (Table 2).

**Table 2:** Recommended health conditions for routine health check-ups for adolescents

Health condition	Specific type of health condition requiring check-ups according to stakeholders’ recommendations	Groups of stakeholders recommending the specific health condition
Non-communicable diseases	High blood pressure^*, ***£, ¥, $, #***^and diabetes^***£, ¥, $, #***^	• Government departments in the Ministry of Health (MoH) /Education (MoE) • Non-Governmental Organisations (NGOs)/Community-Based Organisations (CBOs) • Public and private schools
	Metabolic risk factors (overweight and obesity) ^*, ***£, ¥, $, #***^	• Government departments in the MoH/MoE • NGOs/CBOs
Physical disabilities	Impaired hearing and vision^*, ***£, ¥, $, #***^	• Government departments in the MoH/MoE • Public schools • NGOs/CBOs
Common mental health problems	Depression, anxieties, and alcohol and substance use^***£, ¥, $, #***^	• Government departments in the MoH/MoE • NGOs/CBOs • Public and private schools
Reproductive health related problems	HIV infection and other Sexually transmitted infections^*, ***£, ¥, $, #***^	• Government departments in the MoH/MoE • NGOs/CBOs • Public and private schools • Health facilities
	Unintended teenage pregnancy^*, ***£, ¥, $, #***^	• Government departments in the MoH/MoE • NGOs/CBOs
Other communicable diseases	Parasitic infections (worms), and typhoid^*, ***£, ¥, $, #***^	• Public schools • Government departments in the MoH/MoE
	Malaria ^*, ***£, ¥, $, #***^	• Government departments in the MoH/MoE • NGOs/CBOs • Public and private schools
	Tuberculosis^*, ***£, ¥, $, #***^	• Government departments in the MoH/MoE • NGOs/CBOs • Public and private schools
	Covid-19 ^***£, ¥, $, #***^	• Government departments in the MoH/MoE • Public schools
Hygiene related health problems	Oral and dental health problems, fungal infections and urinary tract infections^*, ***£, ¥, $, #***^	• Government departments in the MoH/MoE • NGOs/CBOs • Public schools

^*^ *Services available in Mwanza city at primary health facilities;* ^***£***^ *services available in Mwanza city at district hospitals;* ^***¥***^ *services available in Mwanza city at referral general hospitals;* ^***$***^ *services available at city tertiary hospitals;* ^***#***^ *services available in Mwanza city at private facilities*

#### Non-communicable diseases

Stakeholders recommended check-ups for two broad categories of NCDs: high blood pressure and diabetes, and metabolic risk factors (overweight and obesity).

High blood pressure and diabetes: Many stakeholders from government departments and a few from NGOs/CBOs and schools proposed to look for high blood pressure and diabetes as part of routine health check-ups. According to some respondents, some adolescents have high blood pressure and diabetes, but due to lack of routine check-ups and testing they are neither aware of their health status, nor of common modifiable risk factors for these common non-communicable conditions.

*“R: ...nowadays there are adolescents who are already at risk for diabetes due to lifestyle and diet, particularly those in urban areas...”***IDI 09, a stakeholder from an NGO/CBO**

Metabolic risk factors (overweight and obesity): Many stakeholders from NGOs/CBOs and some from government departments groups proposed anthropometric measurements as part of the check-ups. Stakeholders viewed weight and height as important health indicators that should be included in the proposed check-ups. They thought the prevalence of being overweight or obese was increasing rapidly among adolescents due to an unhealthy lifestyle, particularly an unhealthy diet and lack of physical activity. A stakeholder from a government department thought that the problem was more common among adolescents from high socio-economic status families, some of whom may be attending private English medium schools.

In contrast, a stakeholder from a public health facility felt that there was no need for routine check-ups of blood pressure and diabetes for adolescents, and instead recommended giving health education to help increase awareness about NCDs. This stakeholder argued that if adolescents were made aware of NCDs, it would be sufficient to protect them from the diseases during this phase of their development and also in adulthood.

*“****R***:*....If people were taught about non-communicable diseases while they were still adolescents, we would not be living our lives with these illnesses....Therefore, for other non-communicable diseases such as diabetes and others, only education should be provided.....”***~ IDI02, a stakeholder from a public health facility**

#### Physical disabilities (visual and hearing impairments)

Many stakeholders from government departments and public schools and a few from NGOs proposed routine health check-ups for the detection of visual and hearing impairments. They stated that these impairments could negatively affect adolescent’s learning at school and contribute to poor educational outcomes. They pointed out that these conditions, if not checked and treated at an early stage, could lead to severe learning and employment problems as adolescents grow older. A stakeholder from the government department wished the country had programs to capture visual and hearing impairments at an early age.

*“****R***: ...*we teach so many children who experience hearing and visual impairments.... their sight should be checked. When you are writing on a blackboard there is a child who stands up and comes too close in order to be able to see what is written on the blackboard. So, these kinds of children have challenges but have not been screened...”****~*IDI02, a stakeholder from a public school**

##### “R1

…*I have come to realise that as a country, we were supposed to have a program that captures visual and hearing impairment at an early age…unfortunately some of these problems get complicated as the age increases. Therefore, a person can suffer from hearing problems that could have been prevented if it was intervened at an early stage, but there is no program…So, if a parent is not careful, they may notice the problem when it has progressed to a stage that you cannot help the child/adolescent anymore…”***~ IDI09, a stakeholder from government departments**.

#### Common mental health problems (depression, anxieties, and alcohol and substance use)

Many stakeholders from government departments and NGOs/CBOs and some from schools proposed screening for mental health problems, particularly depression and anxiety, alcohol and substance use. They reported that mental health problems were exacerbated by adolescents’ lack of social support, and by the physical health and economic challenges which they face in their daily lives. As a result, adolescents resort to abusing alcohol and other substances as strategies of coping with these problems.

*“****R***: *...mental health..., that’s a massive one among adolescents...You just have to include mental health screening...I think there is still a lot of stigma...I think young people don’t really know who to turn to when they are struggling with making sense of the world...You are in a situation like this one with Covid [Covid-19] and everything that is going on...everything is just a bit up in the air. So I guess that everybody can use a bit more psychological support and I think it is a critical time for adolescents because if they can’t talk about it openly or if they can’t say “hey I am struggling” they will find other ways to cope. And how do you cope as a young person? You know you can do drugs, you can drink, you can do crazy things...So that’s why I mentioned mental health...”***~ IDI12, a stakeholder from an NGO/CBO**

#### Reproductive health related problems

Many stakeholders recommended including checks for HIV infection and other STIs in routine health check-ups. Only several stakeholders recommended pregnancy test to be included.

HIV and other STIs: Stakeholders from government departments reported that adolescence was a period of exploration including engaging in high-risk sexual activities, smoking and substance use. They cited that the increasing number of teenage pregnancies indicates that adolescents were sexually active but not using condoms, and thus were at risk of HIV and other STIs as well as unintended pregnancies. Stakeholders from NGOs/CBOs reported that female adolescents were at most risk of HIV and other STIs because they are more likely to be involved in sexual relationships with older men who put them at risk of infection. Stakeholders from health facilities reported that adolescents engage in unsafe sexual activities due to limitations to accessing correct sexual and reproductive health information.

*“****R***: *...sexually transmitted infection should be given priority [for routine check-ups]...nowadays when you investigate, most of children aged between 14 to 19 years have STIs...how did they acquire the infection? It is due to lack of correct education/information about reproductive health...”****~*IDI02**, ***a stakeholder from a p*ublic health facility**

A stakeholder from a government department added that adolescents who are not sexually active may have been infected with HIV through mother-to-child transmission, but due to lack of routine check-ups and testing they may not be aware of their health status.

*“****R***: *...but they should also be provided with HIV testing...at this age of 10–19 years you may find that a child [adolescent] has never engaged herself/himself into sexual activities and never had sex but was born with HIV...therefore if she/he understands her/his health status earlier enough and starts treatment at an early stage, it is easy to continue to boost her/his body immunity, and live healthy”***~ IDI04, a stakeholder from a government department**

Unintended teenage pregnancies: Several stakeholders from NGOs/CBOs and government department recommended including testing for unintended teenage pregnancy as part of the check-ups. A stakeholder from a government department noted that pregnancy tests should be preceded by sexual and reproductive health education and awareness assessment.

*“…they can undergo pregnancy test, but first she should know how one gets pregnant… so we go back to education…first she should be tested for her understanding and then get pregnancy test…”* ~ **IDI08 a stakeholder from a government department**

#### Other communicable diseases

Many stakeholders recommended to routinely screen for three other important communicable conditions: parasitic infections (especially worms), typhoid, malaria, and tuberculosis. Only a few stakeholders proposed including screening for Covid-19.

Worms and typhoid: Many stakeholders from schools and some government departments proposed including screening for “worms and typhoid” as part of the routine check-ups. Worm infections and typhoid were reported to be common among adolescents due to a range of reasons including lack of safe and clean drinking water, and adolescents’ eating, swimming and playing behaviours in unhygienic environments.

*“****R***: *worms...they should be checked for those diseases because clean and safe drinking water is still a challenge in our community....we have clean water at our school but not at students’ homes. When you ask students, you find that at home they drink unboiled water. They also play in water especially during the rainy season because we are close to rivers....many students also eat from food vendors whose cleanliness management is a problem...”****~ IDI04, a stakeholder from a public school***

In addition, stakeholders from government departments mentioned that worm infections caused anaemia which in turn affected children’s academic performance at school. They pointed out that adolescents with worm infections often complained about stomach pains and those with a high worm burden were often inactive at school.

*“R: ...worm infection is an important condition to be screened for because once adolescents become anaemic they lose concentration…high density of worms just cause them to sleep in the class...”***IDI13, a stakeholder from a government department**

Malaria: Stakeholders from schools, government departments and NGOs/CBOs proposed including malaria in the check-ups in order to prevent severe medical complications associated with this condition. Malaria was seen to be a major public health problem in Tanzania, and relatively common among adolescents who often report symptoms suggestive of this condition at school. This affects their school attendance and overall academic performance.

*“R: …Malaria is still a big problem…the uptake of malaria prevention strategies is still low”* **~ IDI03, a stakeholder from an NGO/CBO**

*“****R***: *...Malaria should be included in the check-ups...many children come to school in the morning and start complaining that they are sick...”* ~ **IDI02, a stakeholder from a public school**

In addition, stakeholders from all four sampled groups proposed including screening for anaemia as part of the check-ups. They reported that anaemia was often not checked among adolescents. A stakeholder from a government department was concerned with the frequent occurrence of anaemia among adolescents, particularly among girls.

*“****R***: *...another service that I would recommend to be routinely checked is haemoglobin level because many are transitioning into a reproductive phase and according to the 2016 Tanzania Demographic Survey, the percentage of anaemia is still high. I think it is above fifty percent...the problem is also still big among adolescents...”****~*IDI12, a stakeholder from a government department**

Tuberculosis: Some stakeholders from government departments and schools and a few from NGOs/CBOs proposed including screening for tuberculosis as part of routine check-ups but did not explain the rationale for proposing it.

Covid-19: Only two stakeholders (one from a government department and one from a public school) proposed that screening for Covid-19 infection should be part of routine check-ups. They reported that Covid-19 screening would help to track Covid-19 cases and prevent further spread of the infection. They also pointed out that schools are not equipped with the required facilities for such screening despite of the increased risk of transmission among school-going adolescents due to the large number of students per classroom.

*“****R***: *...and currently there is Covid-19...this should also be included in the check-ups...we do not have any equipment for testing for the virus here and classrooms are overcrowded with students, you cannot identify who is infected or who is not” ~***IDI02, a stakeholder from a public school**

#### Hygiene related health problems

Some stakeholders from NGOs/CBOs and a minority of those from public schools and government departments proposed to include screening for hygiene related problems (oral and dental diseases, fungal and urinary tract infections) in the routine check-ups. The stakeholders reported that poor sanitation, adolescent’s poor eating and hygiene practices contributed to such hygiene related health problems.

*“****R***: *…teeth off course, young people eat sweets, biscuits, and their teeth decay…”***~ IDI01 a stakeholder from an NGO/CBO**

*“****R***: *… but also UTI [should be screened]… our toilets are not very good, sometimes there are no water in some of the toilets…” ~***IDI01 a stakeholder from a public school**

*“****R***: *… speaking of 10 to 19 [years old adolescents]… genital fungus… Yes, I would recommend that they are included… many of them have fungus… they wash their underwear and wear them while they are not well dried…” ~* **IDI08 a stakeholder from an NGO/CBO**

#### Health interventions proposed to be combined with health check-ups

Stakeholders recommended two types of health interventions to be combined with health check-ups for three broad categories of health conditions: mental health problems, reproductive health related problems, and hygiene related problems. The proposed interventions included counselling/education, and provision of family planning information. The stakeholders did not recommend such interventions to be combined with screening for other proposed health conditions.

Counselling/education: A few stakeholders from government departments, NGO/CBOs and schools proposed offering counselling or health education to both boys and girls during the routine check-ups for mental health problems, reproductive health problems (HIV/AIDS, prevention of teenage pregnancy and menstrual health), and hygiene related health problems. The stakeholders noted that counselling or education would help adolescents to understand the problems and the reasons for the routine check-ups.

*“****R***: *...mental health…advice and counselling…especially helping young people [adolescents] to understand and recognise themselves because sometimes they are unable to know the changes that are happening in their bodies…they do many things due to false beliefs…” ~***IDI06, a stakeholder from an NGO/CBO**

Provision of family planning information: Many stakeholders from NGOs/CBOs, one representative from a government department, and one from a school, proposed the provision of family planning information to prevent unplanned teenage pregnancies among adolescents to be included during reproductive health related problems check-ups. They mentioned that unplanned pregnancies or worries about becoming pregnant among sexually active school-going adolescents affected their school performance. They also added that pregnancy among school-going adolescents limited their chances of achieving future goals as the education policy in Tanzania does not allow them to continue with school once they become pregnant.

*“****R***: *You have to at least do family planning counselling....you can’t have a conversation about HIV and sex without talking about family planning. And family planning comprises a lot of issues because if she gets pregnant right now, she is thrown out of school, that’s a matter of policy. So if she is kicked out of school then you know we normally say the odds of that person to achieve her future goals become limited.....so for me that’s why family planning is a priority.....”***IDI12, a stakeholder from an NGO/CBO**

### Proposed venues for health check-ups

Three potential venues were proposed for conducting health check-ups. This included youth-friendly health facilities, schools, and suitable places within the community. Some stakeholders also proposed outreach services as an alternative or addition to these three venues (Table 3). No stakeholder recommended to provide separate venues for girls and boys.

**Table 3:** Proposed venues for health check-ups

Venues for the health check-ups	Stakeholders recommended the venue to be suitable for:	Groups of stakeholders recommending the venue
Youth-friendly health facilities	Health conditions requiring a high level of confidentiality (such as mental health and reproductive health related problems)	• Public health facilities • Government departments in the Ministry of Health (MoH) /Education (MoE) • Non-Governmental Organisations (NGOs)/Community-Based Organisations (CBOs) • Public and private schools
At schools	School-going adolescents	• NGOs/ CBOs • Public and private schools • Government departments in the MoH /MoE
Convenient locations in the community	Older/out-of-school adolescents and those who are unable to visit health facilities for various reasons	• NGOs/ CBOs • Government departments in the MoH/MoE • Public schools
Outreach services	Those who do not wish to visit health facilities when they are not sick	• NGOs/ CBOs • Private schools

#### Youth-friendly health facilities

Many stakeholders from public health facilities, and a few from NGOs/CBOs, government departments, and private schools proposed conducting check-ups at youth-friendly health facilities. Such venues were noted to be suitable for health conditions requiring a high level of confidentiality such as sexual and reproductive health services and treatment services for substance abuse.

*“****R***: *I wish reproductive health services and substance abuse [“vilevi”] to be done at youth-friendly health facilities because check-ups for substance abuse are not reported [disclosed]. You will fail to do it at school because if a child is found to be using substances he/she usually doesn’t get treated, he or she gets punished. Therefore, such screenings are very risky for the screened person, they should be done at health facilities to help the adolescent…”***~ IDI02, a stakeholder from a government department**

Stakeholders thought that youth-friendly health facilities were widely available in Tanzania, providing a range of services in reproductive health, cancer screening and treatment, and mental health. They suggested integrating the check-ups into existing health facilities instead of duplicating efforts by creating new centres outside the mainstream healthcare system.

##### “R

…. *the government has already invested a lot in issues related to youth-friendly services. Just integrate….we should not start to re-invent the wheel….we normally incur a lot of loss in reinventing the wheel…. we should build on what we have*. *For the services that seem to be dormant, let’s revamp them….”***~ IDI05, a stakeholder from a government department**.

#### At schools

Schools were proposed as suitable venues for adolescent health check-ups by many stakeholders from NGOs/CBOs and schools and some from government departments. However, stakeholders did not indicate specific times when check-ups could be conducted without disrupting school activities. The school environment was thought to be both suitable and convenient for reaching school-going adolescents.

“***R***: ....*most of 10 to 14 years adolescents are in primary schools, and of course, most of 15 to 19 years are in secondary schools.... Schools are places where you can get many people, especially adolescents...”****~*IDI08, a stakeholder from an NGO/CBO**

Although the school environment was considered favourably, two main concerns were raised. Firstly, stakeholders thought that the venue was not suitable for health conditions that required a high degree of confidentiality such as HIV infection. Secondly, using this venue would lead to missing adolescents who have left school.

*“****R***: *....the only problem is lack of confidentiality and the ability to deal with challenges that happen at schools....So, if you follow this population at school, the health check-up exercise becomes more relaxed than conducting it at the health facilities....So, school is an ideal [venue] despite that there will be some challenges on how to deal with people who reacted to their test results and also a challenge regarding the possibility of missing (adolescents who are) out of school and school drop-outs....So, everything [venue] has cons and pros, so it very much depends on how sensitive the issue [the health condition] is....”****~*IDI11, a stakeholder from a government department**

#### Convenient locations in the community

Many stakeholders from public schools and NGOs/CBOs and some from government departments proposed conducting the check-ups at convenient locations within the community. They said such venues were suitable for reaching adolescents who have left school. They proposed that such venues should be located at convenient places within the community and check-ups should be conducted at a time agreed by both the community and implementers. Stakeholders were in favour of this type of venue in order to include those who are unable to visit health facilities due to various reasons.

“***R***: ….*we have a challenge of youth [adolescents] not showing up at health facilities when they are feeling well. This has even led us to mostly implement our current program through outreach services in the community…. Therefore, if a community-based approach is possible, let’s follow the adolescents in the community in an environment which is friendly to them…. that way we can be able to reach many and help them….”****~*IDI07, a stakeholder from an NGO/CBO**.

#### Outreach services

Lack of funds for transport or payment of services was reported by stakeholders from NGOs/CBOs as potential barriers for adolescents from poor families or hard to reach areas to access services. Therefore, some stakeholders from private schools and a few from NGOs/CBOs proposed that health check-ups should be delivered through outreach services. Stakeholders were in favour of such outreach services as many adolescents were known to avoid health facilities when they are not sick.

*“****R***: *…. For example, the [name withheld] club does not request us to send children [students] at a certain place.... they come and camp at the school....they spend the whole day screening the youth ....Create a team, go and camp somewhere....it becomes easy for people in that area to know that certain services are being provided today....therefore, many people can show up unlike when you call them to come for services [at a health facility]. People tend to ignore such calls but once you bring services closer to them [through outreach] it is easy for them to participate” ~****IDI06, a stakeholder from a private school***

### Proposed mode of delivery of routine health check-ups

Two types of service providers or relevant stakeholders for health check-ups were proposed. These include trained young and adolescents/youth friendly-service providers; and engagement of a range of relevant stakeholders from selected government departments, private sectors, and schools (Table 4).

**Table 4:** Proposed type of service providers

Recommended type of service providers or relevant stakeholders to be engaged in the check-ups	Groups of stakeholders recommending this type of service providers
Trained young and youth friendly-service providers	• Public health facilities • Non-Governmental Organisations (NGOs)/Community-Based Organisations (CBOs) • Government departments in the Ministry of Health (MoH) /Education (MoE) • Private schools
Stakeholders from selected government departments, private sectors, and schools	• Government departments in the MoH /MoE • Public and private schools • Public health facilities • NGOs/CBOs

#### Trained young and adolescent/youth-friendly service providers

Many stakeholders from all groups proposed routine health check-ups to be provided by young people and staff who are adolescent/youth friendly. Stakeholders mentioned that adolescent-friendly services include: (1) confidentiality of clients’ service information; (2) convenient time for adolescents; (3) convenient location for the adolescents; (4) service accessibility; (5) service availability; and (6) youth-friendly, trained service providers.

*“****R***:.... *of course you cannot say we should find a doctor who is 17, 18 years old....but let’s look at those who are compatible with adolescents...the language they speak is that of adolescents...to attract the adolescents to come and access the services, they must be managed by their peers but not adolescents who are as old as themselves, but at least they should be young”* ~ **IDI04, a stakeholder from an NGO/CBO**

*“****R***: *... by youth friendly services I mean there is a special area for youth/adolescents, and there are experts in youth/adolescents services.... ”****IDI03, a stakeholder from an NGO/CBO***

#### Engaging a range of relevant stakeholders

Stakeholders reported that cultural beliefs, taboos and myths related to adolescents’ sexual activities and reproductive health services were common, and could hinder access to the services. To ensure that services are acceptable and sustainable, many stakeholders from government departments, schools, health facilities and a few from NGOs/CBOs proposed that a range of personnel from government departments, NGOs/CBOs, and schools must be involved in the implementation of routine check-ups.

*“****R***: *...there should be a meaningful engagement of the relevant authority mandated with the adolescents whether it is the Ministry of Education or the Ministry of Health or PO-RALG, but also with the specific regions, adolescents themselves, and the community....It can be challenging when you start screening while the community has no sense of what is happening...there is a need to engage the community so that they can understand what we want to do and why we want to do it among adolescents...and by involving the relevant authorities, if they then provide you with the guidance on what to do, I think it can lead into good results...”****~*IDI03, a stakeholder from a government department**

## Discussion

This study showed that all interviewed stakeholders from government departments and non-governmental organisations were supportive of providing routine health check-ups for adolescents. They specifically recommended check-ups for NCDs, physical disabilities, common mental health problems, reproductive health-related problems, communicable diseases, and hygiene-related health problems. Additionally, stakeholders stressed that the check-up services must be adolescent-friendly, and while they should be provided in schools because of the ease of reaching large numbers of adolescents; they should also be provided in community settings to reach older and out-of-school adolescents or through outreach services to reach adolescents who do not see a need to visit health facilities when they are not sick; and lastly, they should be provided in youth-friendly health facilities for health conditions requiring a high level of confidentiality.

The health conditions recommended by stakeholders for the routine check-ups are relevant for adolescents in Mwanza city. The conditions are common in Mwanza city [26–30] and/or are easily, cheaply treated, or the vaccination is provided national wide [31–34]. However, contrary to the potential short-and long-term benefits of routine check-ups, it is worth noting that routine check-ups for adolescents could strain the distribution and use of limited resources in health systems in low- and middle-income countries like Tanzania [35, 36]. Phase two of this program in Mwanza city will include health check-ups for 15 selected health conditions and/or groups of health conditions (anaemia; malaria; high blood pressure; schistosomiasis; psychosocial and mental health; drugs, alcohol, and tobacco use; nutritional disorders; sexual and reproductive health and STI/HIV infection, epilepsy, immunization, oral health, vision, hearing, and physical impairments). The selection of the health conditions was based on three criteria: (1) That the intervention is potentially scalable. It must therefore be feasible for at least city-wide implementation in terms of cost, health worker time, and technical demands. (2) That the intervention design is based on existing scientific evidence, in particular with respect to the sensitivity and specificity of the screening strategies applied and the health effectiveness of resulting actions. (3) That the intervention meets the perceived needs of the adolescents themselves. This will be achieved by using a person-centred design approach to co-creation of the intervention by the research team working with adolescents themselves.

The stakeholder’s recommendation to combine counselling/education and information interventions in the routine check-ups for mental health, hygiene-related problems, and sexual and reproductive health problems is in line with the local and the WHO guidelines for youth-friendly services that require the point-of-service delivery to provide information and education [37, 38]. The stakeholders proposed the interventions for three broad categories of health conditions, but phase two of this program in Mwanza city will combine information and education communication materials in the check-ups with six health messages about mental health; sleeping behaviours; healthy eating; physical activities; oral health; and substance use. Developing and communicating the information and education materials should be age-specific and co-created through a participatory approach with adolescents [39] and submitted to the ministry of education for curriculum alignment confirmation before sharing them in schools. However, the criteria for developing the information and education materials will be further refined during Phase two of this programme.

Many interviewed stakeholders recommended using schools and convenient locations in the community (for older and out-of-school adolescents) as venues for health check-ups. However, we did not receive specific recommendations on how health check-ups could be organised in the communities. Experiences from other community-based interventions, such as those which deployed sports activities in promoting HIV/AIDS health education for at-risk youths, could provide useful lessons for planning check-ups in communities [40]. Delivery of health check-ups in schools and community settings should be considered in the context of other potential challenges, including a low demand for services, shortages of healthcare staff, and limited resources to sustain the services [36, 41, 42]. Schools and community settings have been proposed for adolescent health check-ups in some African countries, with specific recommendations on how the check-ups should be organised [5, 43]. Experience from these countries could be relevant in the context of Tanzania too. However, since Tanzania has adopted a national strategy for inclusive education [44], and due to the absence of regular checkups [4], children and adolescents with unidentified and untreated disabilities are more likely to attend public schools and hence a school-based check-up approach is likely to result in good yield.

Many stakeholders strongly recommended both the service itself (the check-ups) and the venue for these services to be adolescent/youth-friendly. This has also been advocated in various national guidelines and strategies [7, 45] and implemented by some NGOs in collaboration with the Ministry of Health. For example, HIV/AIDS testing, family planning, and client-centred voluntary medical male circumcision services have been implemented within the ongoing adolescent/youth-friendly services program [46–48]. In the phase two of this program, check-ups activities will be provided by trained health workers including clinicians, nurses, laboratory technicians or assistants and counsellors. However, whilst these services seem feasible when the check-ups are conducted as part of specifically funded projects, scaling up beyond these projects may be challenging since only about 30% of health service delivery points in Tanzania meet the national standards for adolescent-friendly health services [7, 49]. This points to a need for more investment in adolescent/youth-friendly services. Furthermore, our findings revealed that providers’ negative attitudes and values around providing reproductive health services to young and unmarried adolescents are likely to hinder adolescents from accessing the services, even if they were available. Therefore, the integration of routine check-ups within existing healthcare facilities needs careful consideration to avoid resistance from the community and providers [41, 50, 51], and it may be more effective to establish stand-alone health check-ups in schools and in community venues that are attractive to adolescents, and staffed by workers who have been carefully selected to be welcoming to adolescents irrespective of the health issues that they want to discuss or the service that they want to access.

The success of any health-related program for adolescents depends on the buy-in of key stakeholders, especially government officials and the target population [52, 53]. Our findings show that stakeholders from government departments and public institutions are generally supportive of the proposed content, venues, and mode of delivery of routine health check-ups. This suggests that the check-ups are likely to be accepted by other stakeholders in the study communities and by the adolescents themselves and their parents/guardians [8] and are likely to increase the demand for adolescent-friendly services in the country. Data on the cost of screening programs are scarce, and good screening practices and the impact and outcome of the programs are poorly reported [4]. Further studies on coverage, cost, and health impact of the check-ups among adolescents are needed to facilitate long-term planning. These issues will be addressed in the second phase of the Y-Check programme. Studies like the Y-Check phase two program are needed. A funded policy also needs to be developed to support adolescents to access the services.

Our findings should be considered in the context of the following strengths and limitations. To the best of our knowledge, this is the first study to report recommendations from government and non-government stakeholders about the content, venue, and mode of delivery for adolescent routine health check-ups in Tanzania. We collected data using interviews carried out in person or through virtual platforms, allowing our team to conduct the study during the Covid-19 outbreak in Tanzania. This is in line with a recent study conducted by our team [54], which showed that using virtual platforms and mobile phones for data collection during restrictions related to the Covid-19 pandemic enhanced safety for both the research team and the study participants, as well as saving time and cost for travel.

We had planned to also engage adolescents, parents/guardians, and teachers in participatory workshops and group discussions, but at the time of this study, face-to-face group discussions were not allowed, and schools were closed due to Covid-19 related restrictions. The research restrictions were imposed at the national level and lasted several months, exceeding the time and funds available for the fieldwork of this project. We still plan to include surveys with adolescents during the pilot-testing of the intervention that the current research has led to [55]. Therefore, we cannot triangulate the findings from the key informant interviews with findings we could have obtained from participatory workshops with these groups.

Further, we interviewed stakeholders from primary schools and therefore we may have missed health conditions affecting older adolescents. We did not include stakeholders from secondary schools because we planned to conduct in-depth interviews with teachers who teach students aged 10–14 years old. Finally, whilst the use of virtual platforms and mobile phones to collect information was inevitable for this study, it limited our ability to use or capture non-verbal communications such as eye prompts and facial and body expressions. Nonetheless, we paid attention to the stakeholders’ laughter, sighs, tone, pitch, and hesitations during the interviews.

In conclusion, this study shows that stakeholders from government departments and non-governmental organisations were supportive of providing routine health check-ups for adolescents and recommended broad categories of health issues to be checked for, venues to be used, and the mode for delivering the services. In light of these findings, we are now planning to conduct an implementation science research project in which we pilot test the actual implementation of adolescent health and well-being check-ups and evaluate their actual acceptability, feasibility, cost, and yield of new conditions diagnosed and successfully treated when linked, where necessary to on-the-spot treatment and/or referral to free health or social services.

## Electronic supplementary material

Below is the link to the electronic supplementary material.

Supplementary Material 1

Supplementary Material 2

## Data Availability

To maintain and protect the respondents’ anonymity and confidentiality, interview audio and/or transcripts will not be made available to the public. However, anonymised data tables can be made available upon request to the MITU scientific director who is also a principal investigator of this study at MITU on the following contact information: Dr. Saidi Kapiga; Email address: Saidi.Kapiga@lshtm.ac.uk; Telephone: +255 28 2,500,019.

## Abbreviations

CBO: Community-Based Organisations
HIV/AIDS: Human Immunodeficiency Virus/ Acquired Immunodeficiency Syndrome
IDIs: In-depth interviews
LMICs: Lower-middle-income countries
LSHTM: London School of Hygiene and Tropical Medicine
MITU: Mwanza Intervention Trials Unit
NCDs: Non Communicable Diseases
NGO: Non-governmental organisations
NIMR: National Institute for Medical Research
SDGs: Sustainable Development Goals
STIs: Sexual Transmitted Infections
PO-RALG: President’s Office, Regional Administration and Local Governments
WHO: World Health Organization
