# Supplementary material for: Routine health check-ups for adolescents in Mwanza City, Tanzania: stakeholders’ recommendations on its content, venue, and mode of delivery
Source: BMC Public Health. 2023 May 30;23:1015. doi: 10.1186/s12889-023-15956-6 (PMC10227790; doi:10.1186/s12889-023-15956-6)
Supplement: Supplementary file 1 — Supplementary Material 1 [file 12889_2023_15956_MOESM1_ESM.pdf]

Y-Check Study Instrument:

Semi-structured interviews with Key Informants

Objectives of the Interview

(Note: The relative importance of each of these objectives will differ between key informants, based on their organization and specific expertise)

1. Obtain factual information on what health and relevant social services are already in place for adolescents.
2. Gather information on key adolescent health indicators (eg. prevalence of various health conditions by age and sex).
3. Obtain the opinions of key informants on the feasibility, frequency, age range and services that they think should be provided during routine health check-ups for adolescents.
4. Collect the opinions of key informants on how best such routine health check-ups for adolescents might be delivered.

Interview process: Interviews should be tailored to the respondents' area of expertise. The key informant should have been sent the questions below in advance so that they could have the chance to prepare for the interview. The interviewer should obtain informed consent using ICF (Y-CHECK_ICF-Interview/Wshop(KI)_v2.0_21Nov2019) > which includes asking their permission to audio record the interview. If a Key Informant wants to invite one or more of their colleagues to join the interview, that is fine, but each person complete an informed consent form.

The interview should begin with open-ended questions to allow the respondent to provide their perspective without prompting (suggesting potential answers to) them. As the interview progresses, the questions can become more specific to ensure that full use is made of the key informant's relevant expertise.

Guide and script for interviews with key informants

Section 1: Introduction & Informed Consent

See ICF (Y-CHECK_ICF-Interview/Wshop(KI)_v2.0_21Nov2019).

If permission has been granted for audio recording, switch on the recorder. State your name and the date and use a coding system to identify the person being interviewed. Whether or not audio recording is permitted, the interviewing team should write down the key points during the interview. The name and title of the key informant should not be included in reports for privacy reasons.

Section 2: Interview questions, to be adapted to country and key informant context

1. What health care services are provided to adolescents in Mwanza city, Tanzania? Can you provide examples of the types of services provided. Please also elaborate on where these services are most often offered (such as in schools, health facilities, through outreach services, etc.). What organizations provide each service?
2. Would you say that these services are offered equally across groups of adolescents (such as adolescents from high socioeconomic status (SES) families vs. low SES, boys vs. girls or older vs. younger adolescents)? *If there are discrepancies, can you elaborate on why they exist?*
3. If they have not mentioned a school health service, ask: "Is there a school health programme in Mwanza city, Tanzania? If yes, explore further eg. What is included? What organization(s) are involved and for which aspects?
4. What systems are there for referring adolescents from primary care to a specialist? *Please provide examples if possible*. How functional are they?
5. What systems are in place for referring adolescents from schools to primary care or to a specialist? *Please provide examples if possible.* How functional are they?
6. Do adolescents have to pay out-of-pocket for all the health services that they receive? If not, which ones are free? And which are covered by health insurance?
7. Are you aware of any programme that is providing routine health check-ups for adolescents in Mwanza city, Tanzania? *If so, please give details such as: Who is providing the services? To whom? How are they delivered? What do they include?*
8. We are planning to test whether introducing routine health check-ups for adolescents would be a good idea. What do you think about this? *Explore.*
9. If routine health check-ups for adolescents were to be introduced, what do you think they should include? *Why? Explore.*
10. One issue will be to ensure that if an adolescent is found to have a problem that cannot be dealt with on the spot but needs more specialist care, we'll have to decide how to make that feasible for the adolescent. For example, if they are found to need assessment for spectacles or treatment for depression. How do you think we could make this possible?
11. If they don't mention it, "We are considering introducing a voucher system, where the adolescent is given their referral letter along with a voucher that will show to the provider that the project will cover the costs of their care. We will have negotiated the care being offered and the fees for this with the providers in advance. Have you heard of such a voucher system being used in <country>? What do you think about this idea? *Explore.*
12. Assuming services are available, accessible and acceptable to adolescents, are there any reasons why adolescents may still not use them when they need them? Please explain and give examples.

13. Before we end, I would like to ask whether you could please look at the list of health conditions below and tell me whether you would be able to provide prevalence estimates among **10-14-year-old** adolescents for any of the following in Tanzania?

    Is there anyone else who we should talk to in order to get some of these estimates?

	**Health condition**	**Prevalence (%) All /total (10-14)**	**Prevalence (%) Boys (10-14y)**	**Prevalence (%) Girls (10-14y)**
1.	Alcohol			
2.	Tobacco use			
3.	Illicit drug use			
4.	Immunization			
5.	Dental caries, abscesses or oral lesions			
6.	Vision impairment			
7.	Hearing problems			
8.	Mental health disorders			
9.	Obesity/overweight			
10.	Underweight			
11.	Anaemia			
12.	Sexually Transmitted Infections (STIs)			
13.	HIV			
14.	Worm infections			
15.	Injuries			
16.	Suicide attempts			
17.	Diabetes			
18.	Other noncommunicable diseases (Specify) ________________________________ ________________________________			
19.	Disability/chronic illness (specify) ________________________________ ________________________________			

14. Similarly, can you provide the prevalence estimates among 15-19-year-old adolescents for any of the following in <country>?

Is there anyone else who we should talk to in order to get some of these estimates?

	**Health condition**	**Prevalence (%)**<br><br>**All /total (15-19)**	**Prevalence (%)**<br><br>**Boys (15-19y)**	**Prevalence (%)**<br><br>**Girls (15-19y)**
1.	Alcohol			
2.	Tobacco use			
3.	Illicit drug use			
4.	Immunization			
5.	Dental caries, abscesses or oral lesions			
6.	Vision impairment			
7.	Hearing problems			
8.	Mental health disorders			
9.	Obesity/overweight			
10.	Underweight			
11.	Anaemia			
12.	Sexually Transmitted Infections (STIs)			
13.	HIV			
14.	Worm infections			
15.	Injuries			
16.	Suicide attempts			
17.	Diabetes			
18.	Other noncommunicable diseases (Specify)<br>____________________________<br>____________________________			
19.	Disability/chronic illness (specify)<br>____________________________<br>____________________________			

15. Are you aware of any validated tools being used in your <country>to screen for the major health issues listed above?

For example, use of the PHQ-9 for screening for depression or the Alcohol, Smoking and Substance Involvement Screening Test (ASSIST) for substance use, applications for testing hearing loss, etc. Also, please comment on how commonly these tools are used in the country.

16. Finally, do you have any further suggestions for the content or delivery of the planned health check-ups intervention as we move forward?
