# Supplementary material for: Routine health check-ups for adolescents in Mwanza City, Tanzania: stakeholders’ recommendations on its content, venue, and mode of delivery
Source: BMC Public Health. 2023 May 30;23:1015. doi: 10.1186/s12889-023-15956-6 (PMC10227790; doi:10.1186/s12889-023-15956-6)
Supplement: Supplementary file 2 — Supplementary Material 2 [file 12889_2023_15956_MOESM2_ESM.pdf]

Kitendea kazi cha utafiti wa Y-CHECK

Mahojiano na watoa taarifa maalumu

Malengo ya mahojiano

(Kumbuka: Umuhimu wa kila moja ya malengo haya utatofautiana kati ya watoa taarifa maalumu, kulingana na taasisi wanazofanyia kazi na utaalamu wao)

1. Kupata taarifa za uhakika kuhusu huduma za afya na za kijamii zinazotolewa kwa vijana.
2. Kukusanya taarifa kuhusu viashiria muhimu vya afya za vijana (kwa mfano: ukubwa wa matatizo mbalimbali ya afya kwa umri na jinsia).
3. Kupata maoni ya watoa taarifa muhimu kuhusu uwezekano wa kutoa huduma ya uchunguzi wa afya kwa vijana na itolewe mara ngapi, katika umri gani, na huduma gani zitolewe.
4. Kukusanya maoni ya watoa taarifa muhimu kuhusu namna bora ya kutoa huduma ya uchunguzi wa afya kwa vijana.

Mchakato wa mahojiano: Mahojiano yafanyike kulingana na utaalamu/eneo la ubobezi la mhojiwa. Mtoa taarifa maalum atakuwa ametumiwa maswali (yaliyoainishwa hapo chini) kabla ya haya mahojiano ili apate muda wa kujiandaa. Mhojaji apate idhini ya maandishi ya mtoa taarifa maalumu kwa kutumia formu *(Y-CHECK_ICF-Interview/Wshop(KI)_v2.0_21Nov2019***)** ambayo inajumuisha kuomba idhini ya kurekodi sauti wakati wa mahojiano. Kama mtoa taarifa maalumu atapenda kumualika mwenzake mmoja au zaidi, hiyo ni sawa lakini kila mmoja wao ajaze fomu ya idhini kushiriki.

Mahojiano yaanze na maswali ya wazi (open ended questions) ili kumpa mhojiwa nafasi ya kutoa mtizamo wake bila kumuongoza kwenye majibu. Kadiri mahojiano yatakavyokuwa yakiendelea, maswali yanaweza kuwa na malengo mahsusi ili kuhakikisha tunatumia vizuri utaalamu na uzoefu wa mhojiwa.

Mwongozo wa mahojiano na watoa taarifa maalumu

Sehemu ya 1: Utangulizi & Kuomba idhini ya ushiriki

Tizama fomu namba: Y-CHECK_ICF-Interview/Wshop(KI)_v2.0_21Nov2019

Washa kifaa cha kurekodi sauti kama ruhusa imetolewa. Taja jina lako, tarehe na tumia utaratibu mahsusi kumtambulisha mshiriki anayehojiwa bila kutaja jina lake halisi. Bila kujali kama sauti inarekodiwa au hairekodiwi, watafiti waandike mambo muhimu yanayozungumzwa wakati wa mahojiano. Majina na cheo cha mtoa taarifa maalumu visiwekwe kwenye ripoti kwa sababu za kutunza siri na faragha.

Sehemu ya 2: Maswali yanaweza kubadilishwa kulingana na mazingira ya nchi au muktadha wa mtoa taarifa maalumu.

1. Ni huduma zipi za afya hutolewa kwa vijana katika jiji la Mwanza, Tanzania? Unaweza kutoa mfano wa aina za huduma zitolewazo. Pia, tafadhali fafanua huduma hizi hutolewa wapi (je ni shuleni, vituo vya afya, kupitia huduma za afya zinazopelekwa mtaani (outreach services) n.k.) Ni taasisi gani hutoa hizo huduma?

2. Je, unaweza kusema kwamba huduma hizi hutolewa sawa kwa makundi tofauti ya vijana (kwa mfano, vijana kutoka tabaka la juu kiuchumi na wale wa tabaka la chini, wavulana na wasichana, vijana wakubwa (miaka 15 – 19) na wadogo (miaka 10 – 14)?*. Ikiwa zipo tofauti, unaweza kueleza ni kwa nini kuna tofauti?*

3. **Kama hawajataja huduma za afya mashuleni, uliza**: " Je kuna mpango wa huduma za afya mashuleni katika jiji la Mwanza, Tanzania?. Kama ndiyo, dodosa zaidi kufahamu kwa mfano ni huduma zipi hutolewa?, ni taasisi gani zinahusika na ni kwa namna gani?

4. Je, kuna mfumo mahsusi wa rufaa kwa vijana kutoka kwenye huduma za afya za msingi kwenda kwenye huduma za madaktari bingwa?, *tafadhali toa mfano kama inawezekana.*

Je, mfumo huo unafanya kazi?

5. Je, kuna mfumo mahsusi wa rufaa kwa vijana kutoka mashuleni kwenda kwenye huduma za afya za msingi au kwenda kwenye huduma za hospitali (wilaya, mkoa au rufaa)? *Tafadhali toa mfano kama inawezekana.*

Je, mfumo huo unafanya kazi?

6. Je, vijana hutakiwa kulipia huduma za afya wanazopata kutoka mfukoni mwao?

Kama hapana, ni huduma zipi hutolewa bure? Na ni zipi hulipiwa na bima ya afya?

7. Je, unafahamu kama kuna programu yeyote inayotoa huduma ya uchunguzi wa afya kwa vijana katika jiji la Mwanza, Tanzania?*: **Kama ndiyo,** tafadhali tupatie taarifa za kina zaidi, kwa mfano:*

Ni nani anatoa huduma hizo?

Huduma hizo hulengwa kutolewa kwa nani?

Huduma hizo zinatolewaje?

Huduma zenyewe ni zipi?

8. Tunataka kufanya majaribio kuona kama mpango wa utoaji wa huduma ya uchunguzi wa kawaida wa afya kwa vijana litakuwa ni wazo zuri. Una maoni gani kuhusu wazo hili?

Dodosa

9. Kama mpango wa uchunguzi wa kawaida wa afya za vijana ukianzishwa, ni huduma zipi zitolewe?

Kwa nini? ***Dodosa***

10. Jambo moja, litakuwa kuhakikisha kwamba, kama kijana amekutwa na tatizo ambalo haliwezi kutibika mara moja na hivyo anahitaji huduma za hospitali kubwa zaidi (wilaya, mkoa au rufaa) - tutalazimika kuamua ni namna gani vijana wahitaji watawezeshwa kupata huduma hizo? Kwa mfano, kama wameonekana wanahitaji kuchunguzwa kama wanahitaji miwani au matibabu ya huzuni. Unadhani nini kifanyike kuwawezesha?

11. **Kama wasipoongelea mpango wa malipo kwa vocha**, "tunafikiria kuanzisha mfumo wa malipo kwa vocha, ambapo kijana atapewa barua ya rufaa pamoja na vocha inayomwonyesha mtoa huduma ya afya kwamba mradi wa utafiti utalipia gharama za matibabu. Tayari tutakuwa tumekubaliana na watoa huduma za afya kuhusu huduma zitakazotolewa na gharama zake.

Je ulishawahi kusikia utaratibu wa malipo kwa vocha ukitumika hapa Tanzania?

*Una maoni gani kuhusu wazo hili? **Dodosa**.*

12. Mathalani huduma za uchunguzi wa afya kwa vijana zipo, zinafikika na zinakubalika, je kuna sababu nyingine zitakazowafanya vijana wasiweze kuzitumia wakati wanapozihitaji?

Tafadhali fafanua na utoe mifano

13. Kabla hatujamaliza, Ningependa kukuomba utizame orodha hapo chini na uniambie kama unaweza kutoa makisio ya ukubwa wa kila moja ya masuala ya kiafya miongoni mwa vijana wenye umri wa miaka 10 – 14 hapa Tanzania?

Je, kuna mtu mwingine tunayeweza kuzungumza naye ili kupata baadhi ya hayo makisio?

	**Magonjwa au viashiria vinavyohusishwa na matatizo mbali mbali ya kiafya**	**Makisio (%)**<br><br>**Wote/jumla (miaka 10-14)?**	**Makisio (%)**<br><br>**Wavulana (miaka 10 -14)**	**Makisio (%)**<br><br>**Wasichana (miaka 10 -14)**
1.	Matumizi ya pombe			
2.	Matumizi ya tumbaku			
3.	Matumizi ya dawa za kulevya			
4.	Chanjo			
5.	Kuoza meno na vidonda mdomoni			
6.	Uoni hafifu			
7.	Matatizo ya kusikia			
8.	Matatizo ya afya ya akili<br>(Taja)________________________________<br>____________________________________			
9.	Unene			
10	Uzito hafifu			
11	Upungufu wa damu			
12	Magonjwa ya zinaa<br>(Taja)________________________________<br>____________________________________			
13	UKIMWI			

14	Minyoo			
15	Majeraha			
16	Majaribio ya kujiua			
17	Kisukari			
18	Magonjwa mengine yasiyoambukizwa (Taja)________________________________			
19	Ulemavu (Taja) ________________________________			

14. Hivyo hivyo, unaweza kunipatia makisio ya ukubwa wa kila moja ya masuala ya kiafya yaliyotajwa kwenye oroodha hapo chini miongoni mwa vijana wenye umri wa miaka 15 mpaka 19?

Je, kuna mtu mwingine tunayeweza kuzungumza naye ili kupata baadhi ya hayo makisio?

	**Magonjwa au viashiria vinavyohusishwa na matatizo mbali mbali ya kiafya**	**Makisio (%)**<br><br>**Wote/jumla (15-19)**	**Makisio (%)**<br><br>**Wavulana (miaka 15 -19)**	**Makisio (%)**<br><br>**Wasichana (miaka 15 -19)**
1.	Matumizi ya pombe			
2.	Matumizi ya tumbaku			
3.	Matumizi ya dawa za kulevya			
4.	Chanjo			
5.	Kuoza meno na vidonda mdomoni			
6.	Uoni hafifu			
7.	Matatizo ya kusikia			
8.	Matatizo ya afya ya akili (Taja)________________________________ ____________________________________			
9.	Unene			
10	Uzito hafifu			
11	Upungufu wa damu			
12	Magonjwa ya zinaa (Taja)________________________________ ____________________________________			
13	UKIMWI			
14	Minyoo			
15	Majeraha			
16	Majaribio ya kujiua			
17	Kisukari			
18	Magonjwa mengine yasiyoambukizwa (Taja) ___________________________________			
19	Ulemavu (Taja) ___________________________________			

15. Unafahamu dodoso(au vifaa) lolote lililothibitishwa linalotumika hapa Tanzania kupima masuala ya kiafya yaliyoorodheshwa hapo juu?

Kwa mfano, matumizi ya PHQ-9 kwa ajili ya kupima kiwango cha huzuni (depression), au ASSIST kwa ajili ya kupima matumizi ya pombe, sigara na madawa ya kulevya, vipimo vya usikivu hafifu kwa kutumia mifumo ya simu n.k.

Pia, tafadhali toa maoni yako juu ya namna madodoso na vifaa hivi vinavyotumika hapa Tanzania.

16. Mwisho, Kuhusu mpango unaopendekezwa wa uchunguzi wa afya za vijana, kadiri tunavyosonga mbele, je una mapendekezo zaidi kuhusu nini kichunguzwe na namna gani mpango utekelezwe?
